# A Very Uncommon Case of Myxedema Coma: Rediscovery of an Old Presentation

**DOI:** 10.7759/cureus.27177

**Published:** 2022-07-23

**Authors:** Zain AlShanableh, Krista M Wong, Mohammad Haidous, Deema Chakhachiro, Basel Altaqi

**Affiliations:** 1 Internal Medicine, St. Vincent Charity Medical Center, Cleveland, USA

**Keywords:** effusions, levothyroxine, heart failure, hypothyroidism, myxedema coma

## Abstract

Myxedema coma is an extreme manifestation of hypothyroidism. It is characterized by altered mental status and hypothermia. The most common precipitants of myxedema coma include discontinuation of thyroid supplements and infections. Therefore, the mainstay of treatment is IV glucocorticoids and IV levothyroxine. We describe a case of an 81-year-old woman with myxedema coma who developed acute cardiopulmonary failure with associated pericardial and pleural effusions, which are rare manifestations of myxedema coma.

## Introduction

Myxedema coma is a form of severe hypothyroidism characterized by altered mental status and hypothermia. The term myxedema coma is a misnomer as most patients are altered mentally rather than truly comatosed. The mortality rate may be as high as 25-60% [1]. Patients frequently exhibit signs and symptoms of classic hypothyroidism, as well as symptoms that occur as a result of functional decline in multiple organ systems. The most common precipitants of myxedema coma include discontinuation of thyroid supplements and infections [1]. Timely diagnosis requires early clinical suspicion because early treatment with IV glucocorticosteroids and IV levothyroxine is important in reducing the morbidity and mortality rates of this fatal disease [2]. Overt congestive heart failure is rare in the absence of preexisting cardiac disease [3-5]. We describe an 81-year-old woman with a myxedema coma who developed acute cardiopulmonary failure as well as pericardial and pleural effusions. This case was presented as a poster at the "Ohio Chapter/Air Force Chapters Annual Abstract Competition" in October 2020 [6].

## Case presentation

An 81-year-old female with a past medical history of hypertension, transient ischemic attack, dementia, and primary hypothyroidism presented with a one-day history of worsening shortness of breath. She developed altered mental status and became mute the week prior to the presentation. She was unable to tolerate oral intake or consume her medications and subsequently developed facial and lower limb swelling. On examination, the patient was hypothermic with a temperature of 34.7 ℃, alert but disoriented and non-communicative. She was found to have facial swelling, elevated jugular venous pressure, bilateral coarse crepitations on auscultation of the lung, muffled heart sounds, and cool extremities with 3+ pitting edema in bilateral lower limbs. The patient soon developed cardiogenic shock with respiratory failure and was intubated and kept on inotropic support.

Laboratory investigations revealed a significantly elevated thyroid-stimulating hormone (TSH), with low free thyroxine (fT4) and free triiodothyronine (fT3). Random free cortisol was within normal limits. Other labs showed no leukocytosis or bandemia, elevated creatinine, blood urea nitrogen (BUN), alanine transaminase (ALT), aspartate transaminase (AST), and troponin (Table 1). Chest X-ray revealed vascular congestion and cardiomegaly. ECG revealed T-wave inversions in lateral leads and prolonged QT interval. Transthoracic echocardiogram (TTE) revealed an ejection fraction (EF) of 25-30%, dilated cardiomyopathy, and pericardial and left pleural effusion (Video 1). Previous TTE performed less than one year ago for evaluation of lower extremity edema revealed a normal EF.

**Table 1:** Laboratory workup. TSH: Thyroid-stimulating hormone; BUN: Blood urea nitrogen; ALT: Alanine transaminase; AST: Aspartate transaminase.

Laboratory work	Value	Reference range
TSH	102 uIU/mL	0.358-3.74
Free T4	0.11 ng/dL	0.76-1.46
Free T3	<0.5 pg/ml	2.18-3.98
Cortisol	68 ug/dL	AM (7-9am): 5.27-22.45
		PM (3-5pm): 3.44-16.76
WBC	8.2 K/uL	3.9-11
Creatinine	1.94 mg/dL	0.55-1.02
BUN	38 mg/dL	7-18
ALT	136 U/L	13-61
AST	303 U/L	15-37
Troponin	0.06 ng/ml	0.01-0.045

**Video 1:** Transthoracic echocardiogram.

The final diagnosis was a cardiopulmonary failure with myxedema coma. The patient was managed in the ICU with IV levothyroxine, steroids, and appropriate cardiopulmonary support. She gradually regained consciousness, was successfully extubated and was eventually discharged.

## Discussion

Myxedema coma is a form of severe hypothyroidism characterized by a change in mental status and hypothermia [1]. The incidence of myxedema coma follows the same pattern as hypothyroidism and is more common in women. It is associated with a significantly high mortality rate of 25-60% [1]. The term myxedema coma is a misnomer as most patients are altered mentally rather than truly comatosed [1]. 

Myxedema coma is often precipitated by factors such as cold exposure, myocardial infarction, use of sedatives, infection, or discontinuation of thyroid supplements, or it can also occur as a culmination of severe chronic hypothyroidism [1,2]. Our reported patient precipitants included discontinuation of thyroid supplements and cold exposure as the patient presented in December in the city of Cleveland, Ohio. Patients frequently exhibit signs and symptoms of classic hypothyroidism in addition to symptoms resulting from functional decline in multiple organ systems [2]. Physical examination findings include goiter, non-pitting edema, dry skin, hoarse voice, macroglossia, sparse hair, and delayed tendon reflexes [2]. Other clinical features include anemia, hyponatremia, hyperlipidemia, hypoglycemia, elevated creatine phosphokinase, transaminases, and creatinine and respiratory acidosis [2]. In addition, there is sinus bradycardia, low voltage complexes, heart blocks, and prolonged QT on ECG [2].

Cardiovascular manifestations and hemodynamics of myxedema coma include bradycardia, decreased myocardial contractility, diastolic hypertension despite cardiac output reduction, narrowed pulse pressure, and hypotension occurring later in the disease course [3]. Thyroid hormone exerts its effects by reducing peripheral arteriolar resistance via a direct effect on vascular smooth muscles and reduces mean arterial pressure. This reduces systemic vascular resistance, which activates the renin-angiotensin-aldosterone system, leading to enhanced sodium absorption by the kidneys [3]. Thyroid hormone also stimulates contractility of the left ventricle by regulating the expression of several cardiac genes that encode contractile proteins and membrane channels [3]. It also increases the resting heart rate and erythropoietin production. These changes merge to increase RBC mass, blood volume, and preload and reduce afterload, resulting in an overall increased cardiac output [3]. In hypothyroidism, cardiac output may decrease by 30-50% [3]. In addition, pleural and pericardial effusion may be present due to increased vascular permeability [3]. 

Overt congestive heart failure is rare due to solely hypothyroidism and is more commonly seen in patients with preexisting cardiac diseases [4,5]. 

The diagnosis of myxedema coma is initially based upon the history, physical examination, and exclusion of other causes of coma/altered mental status. Therefore, the diagnosis must be highly suspected in any patient presenting with coma/altered mental status and hypothermia. When the diagnosis is suspected, a blood sample should be drawn prior to initiation of treatment for measurement of the following: TSH, fT4, and cortisol [2].

Treatment should be initiated based on clinical suspicion without waiting for laboratory confirmation. The American Thyroid Association recommends that patients receive an empiric dose of IV glucocorticoids, at a stress dose, prior to initiation of levothyroxine therapy [7]. This is given to avoid precipitation of adrenal crisis if the patient is cortisol deficient. Levothyroxine should initially be administered intravenously at a loading dose of 200-400 mcg [7]. Lower doses should be used for patients with coronary artery disease, arrhythmias, small stature, and the elderly. Subsequently, a daily maintenance dose is administered based on weight, with the recommended dose being 1.6 mcg per kilogram per body weight. This dose is reduced to 75% if administered intravenously and until the patient can tolerate levothyroxine orally upon clinical improvement [7]. Treatment also consists of appropriate supportive measures and management of coexisting problems (e.g., infection) [7].

## Conclusions

We present this case to emphasize the importance of early diagnosis and management of a rare and potentially fatal condition. Identifying early signs and symptoms with prompt medical attention is especially important in reducing morbidity and mortality. Myxedema coma should be suspected in elderly women presenting with altered mental status and hypothermia, with or without signs of chronic hypothyroidism. Cardiopulmonary failure is rare in the absence of preexisting cardiac diseases, which makes our reported case of particular interest.
